# Chemotherapy-induced myasthenia gravis presenting as isolated bulbar palsy in a patient with WHO type B3 thymoma: A case report

**DOI:** 10.1097/MD.0000000000050223

**Published:** 2026-08-14

**Authors:** Huiming Xu, Yihua Lai, Rongqiang Shen, Wenshan Zhang, Guoyi Shen, Yi Zhang

**Affiliations:** a Department of Thoracic Surgery, Zhangzhou Affiliated Hospital of Fujian Medical University, Zhangzhou, Fujian Province, China.

**Keywords:** bulbar palsy, CAP chemotherapy, case report, myasthenia gravis, thymoma, WHO type B3

## Abstract

**Rationale:**

Myasthenic crisis rarely manifests exclusively as isolated bulbar palsy in patients with WHO type B3 thymoma undergoing neoadjuvant chemotherapy, often leading to misdiagnosis as chemotherapy toxicity or infection.

**Patient concerns:**

A 55-year-old man with an invasive anterior mediastinal mass developed progressive dysphagia and dysarthria 5 days after the first cycle of CAP (cyclophosphamide, doxorubicin, cisplatin) chemotherapy, raising concerns for acute neurological deterioration.

**Diagnoses:**

Neurological examination localized to bulbar involvement. Elevated anti-acetylcholine receptor antibodies (1.03 nmol/L), a positive ice-pack test, and objective improvement with the neostigmine test confirmed a chemotherapy-induced myasthenic crisis. A core-needle biopsy had previously established a WHO type B3 thymoma diagnosis.

**Interventions:**

The patient was placed nil by mouth with nasogastric decompression and correction of cisplatin-associated hypomagnesemia. Immunomodulation included pulsed methylprednisolone, intravenous immunoglobulin, and oral pyridostigmine. After symptom resolution, he completed a second CAP cycle followed by R0 video-assisted thoracoscopic thymectomy and adjuvant radiotherapy (60 Gy).

**Outcomes:**

Bulbar symptoms resolved completely within 4 days of immunomodulation. At 29 months of follow-up, neither local tumor recurrence nor myasthenia gravis relapse was observed, with excellent performance status.

**Lessons:**

Isolated bulbar palsy can be the sole inaugural sign of chemotherapy-triggered myasthenic crisis. Clinicians should rapidly screen for acetylcholine receptor antibodies in thymoma patients receiving cytotoxic therapy. Integrating baseline serological risk assessment before chemotherapy may prevent life-threatening delays in care.

## 1. Introduction

**Figure 1.**
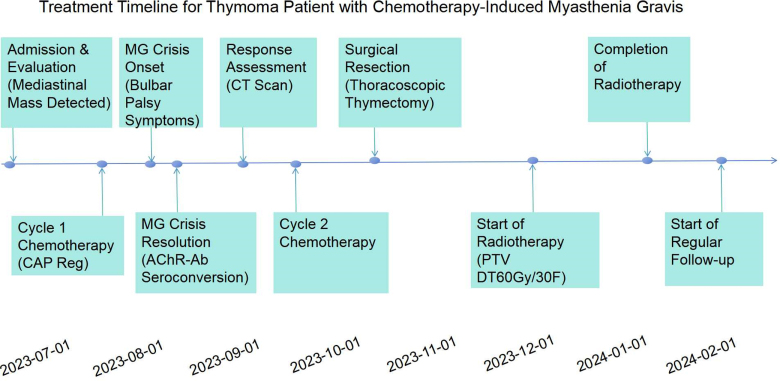
Treatment timeline for a patient with WHO type B3 thymoma and chemotherapy-induced myasthenia gravis. The patient underwent neoadjuvant CAP chemotherapy in July 2023. Five days after the first cycle, he developed a myasthenic crisis presenting exclusively as isolated bulbar palsy. Following successful immunomodulation and symptom resolution, restaging CT showed a near-complete response. He subsequently completed a second cycle of CAP, underwent R0 thoracoscopic thymectomy in October 2023, and received adjuvant radiotherapy (60 Gy) from December 2023 to January 2024. AChR-Ab = anti-acetylcholine receptor antibody, CAP = cyclophosphamide, doxorubicin, cisplatin, CT = computed tomography, PTV = planning target volume.

Myasthenic crisis is uncommon but life-threatening in patients with thymoma, and it may emerge or worsen during perioperative systemic therapy. In the neoadjuvant setting, cranial nerve/bulbar presentations are especially prone to misattribution (e.g., chemotherapy-related mucositis, brainstem stroke, metabolic encephalopathy, or infection), delaying targeted immunomodulation and ventilatory rescue.^[1–3]^ WHO type B3 thymomas are more frequently associated with autoimmune/myasthenic phenomena because of their epithelial predominance and “thymoma niche” antigenic environment.^[4,5]^ We report a case in which isolated bulbar palsy was the inaugural and sole manifestation of chemotherapy-induced myasthenia gravis (MG) crisis after CAP (cyclophosphamide, doxorubicin, and cisplatin) for a B3 thymoma – occurring without glucocorticoid premedication/antiemetics – and outline the multidisciplinary pathway that permitted safe downstaging and R0 resection. The overall clinical course and management timeline are summarized in Figure 1.

## 2. Case report

**Figure 2.**
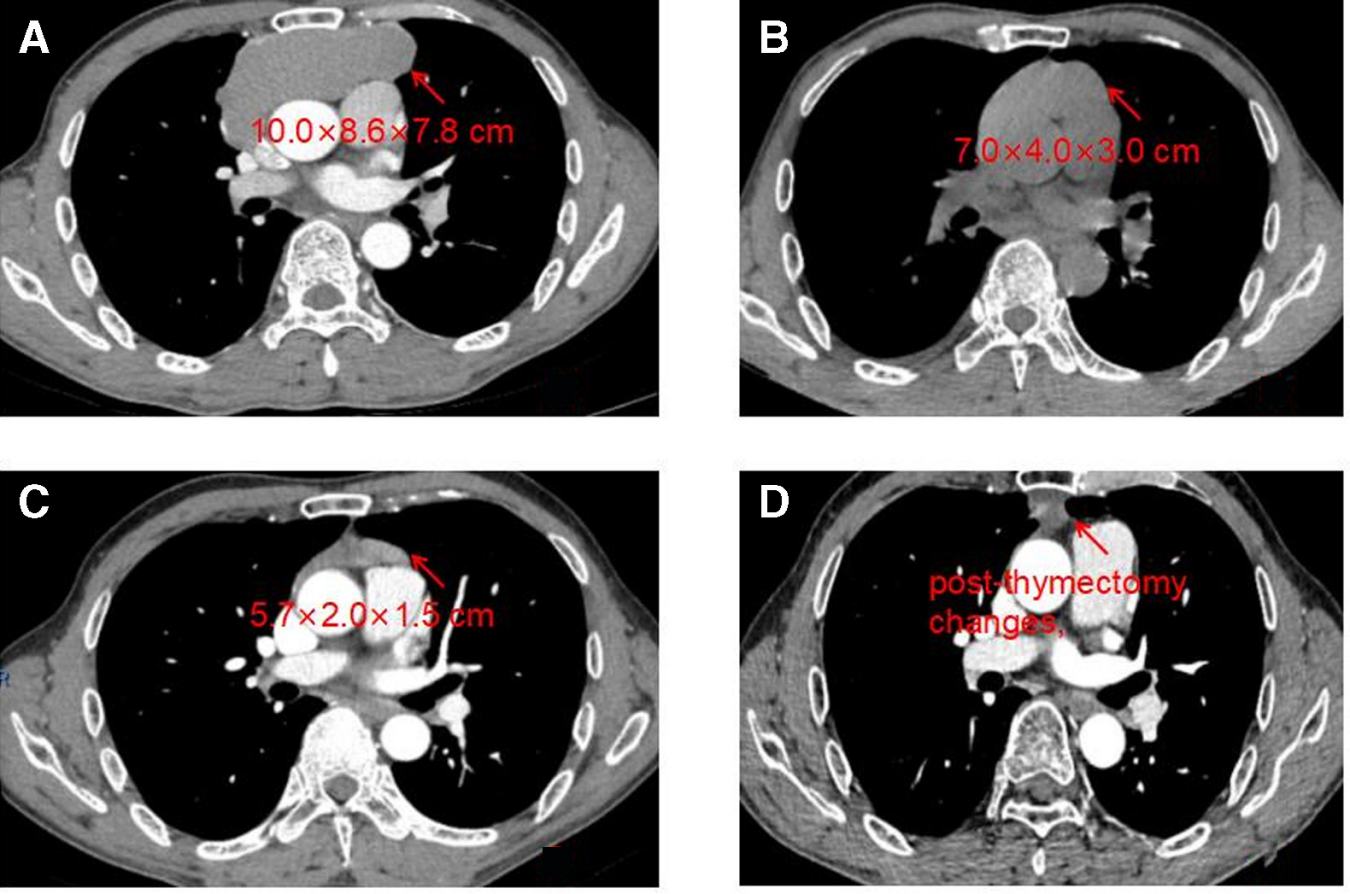
Serial contrast-enhanced CT images. (A) Baseline (July 2023): large anterior mediastinal mass (10.0 × 8.6 × 7.8 cm) with abutment of the left innominate vein/right atrial appendage (red arrow). (B) After 1 cycle of CAP: marked regression to ~7.0 × 4.0 × 3.0 cm (red arrow). (C) After 2 cycles: further reduction to 5.7 × 2.0 × 1.5 cm residual strand/fibrosis (red arrow). (D) Postoperative (3 months): postsurgical changes at the thymectomy site; no local recurrence. CAP = cyclophosphamide, doxorubicin, cisplatin, CT = computed tomography.

A 55-year-old man was referred for an incidentally discovered anterior mediastinal mass. Baseline neurologic examination was normal (no ptosis, ophthalmoplegia, or limb weakness). Contrast-enhanced computed tomography (CT) demonstrated a 10.0 × 8.6 × 7.8 cm heterogeneously enhancing anterosuperior mediastinal mass abutting the left innominate vein and right atrial appendage, consistent with invasive thymoma (Masaoka-Koga stage III).^[1]^ Serial contrast-enhanced CT images are shown in Figure 2, demonstrating the tumor’s dynamic changes. Core-needle biopsy rendered a WHO type B3 diagnosis.^[4]^ Preoperative anti-acetylcholine receptor antibody (AChR-Ab) screening was not performed.

Because of tumor size and vascular adherence, a neoadjuvant strategy was adopted. Day 1 (July 25, 2023) comprised CAP: cisplatin 50 mg/m^2^, doxorubicin 50 mg/m^2^, cyclophosphamide 500 mg/m^2^ intravenous.^[6,7]^ No glucocorticoids were administered for premedication/antiemetics.

On day 5 post-cycle 1, he acutely developed progressive dysphagia (liquid choking), dysarthria, and bulbar fatigability; vital signs and bedside pulmonary mechanics remained initially reassuring. Given the thymoma history, MG crisis was suspected. AChR-Ab was elevated (1.03 nmol/L); the ice-pack test was positive; the neostigmine test produced objective bulbar improvement (with muscarinic side effects requiring atropine). The working diagnosis was chemotherapy-induced MG crisis manifesting as isolated bulbar palsy.^[2,3]^

Management included nil by mouth/nasogastric decompression and correction of cisplatin-associated hypomagnesemia. Immunomodulation comprised methylprednisolone 500 mg daily × 3 days with taper, plus intravenous immunoglobulin 0.4 g/kg/d × 5 days;^[2,3]^ pyridostigmine 60 mg t.i.d. was initiated. Bulbar symptoms resolved within 4 days; AChR-Ab normalized.

Restaging CT (September 7) showed marked regression (7.0 × 4.0 × 3.0 cm; near-complete response-like appearance). The second CAP cycle (September 14) was administered uneventfully. On October 9, he underwent right video-assisted thoracoscopic surgery extended thymectomy + partial pericardiectomy; it partially invaded the left innominate vein. R0 resection was achieved using sharp dissection and primary venorrhaphy (5-0 Prolene). Pathology showed extensive treatment effect (near-complete chemotherapeutic response, grade III–IV).^[4]^ He received adjuvant radiotherapy 60 Gy/30 fractions (November 23–January 3).^[1]^ At 29 months, there is no evidence of recurrence or MG relapse.

## 3. Discussion

This case makes 3 practical points for thoracic surgeons.

First, bulbar-only onset is a real, treacherous myasthenic crisis phenotype. Upper airway/bulbar muscles tolerate neuromuscular junction compromise poorly, yet patients may initially maintain “acceptable” respiratory parameters, creating a false sense of security. Sudden-onset bulbar symptoms during/after thymoma chemotherapy should trigger rapid AChR-Ab testing ± bedside ice-pack/neostigmine challenge rather than defaulting to gastroenterologic or toxic explanations.^[2,3]^

Second, glucocorticoids are not a prerequisite trigger. Because we used zero steroids, this crisis implicates alternative drivers: cytotoxic tumor lysis/release of AChR-type antigens amplifying autoimmunity around the thymoma niche^[4,5]^ and cisplatin-related electrolyte loss (especially hypomagnesemia) lowering the safety margin at the neuromuscular junction.

Third, this case highlights the need for protocolizing pre-chemotherapy autoimmune screening in patients with thymoma. We recommend integrating pre-chemotherapy AChR antibody testing – supplemented by muscle-specific kinase and other relevant autoimmune panels when clinically indicated – as a brief, standardized safety checkpoint. Rather than precluding curative oncologic treatment, this approach enables proactive staging of perioperative monitoring and supportive resources, including hydration and electrolyte correction protocols, early neurology standby consultation, and a low threshold for converting oral anticholinesterase therapy to the parenteral route should neuromuscular symptoms arise.

These findings argue for the integration of baseline AChR-Ab screening prior to neoadjuvant chemotherapy in thymoma patients. Identifying seropositive individuals – particularly those with WHO type B3 histology – enables proactive perioperative monitoring and may preclude life-threatening crises through timely immunomodulation.

## Acknowledgments

The authors would like to thank the patient for his cooperation and consent for publication.

## Author contributions

**Investigation:** Huiming Xu.

**Data curation:** Yihua Lai, Rongqiang Shen, Wenshan Zhang, Guoyi Shen.

**Resources:** Yihua Lai, Guoyi Shen.

**Writing – original draft:** Huiming Xu, Yihua Lai, Rongqiang Shen, Wenshan Zhang, Guoyi Shen, Yi Zhang.

**Writing – review & editing:** Huiming Xu, Yihua Lai, Rongqiang Shen, Wenshan Zhang, Guoyi Shen, Yi Zhang.
